# Challenges and responses of malaria elimination in a high-endemic area along the Thai-Myanmar border: A health systems perspective

**DOI:** 10.1371/journal.pgph.0006286

**Published:** 2026-04-09

**Authors:** Sayambhu Saita, Daniel M. Parker, Kritsana Suk-uam, Suparat Phuanukoonnon, Kasama Pooseesod

**Affiliations:** 1 Faculty of Public Health, Thammasat University, Lampang, Thailand; 2 Thammasat University Research Unit in One Health and Ecohealth, Thammasat University, Pathum Thani, Thailand; 3 Department of Population Health and Disease Prevention, University of California Irvine, Irvine, California, United States of America; 4 Department of Epidemiology & Biostatistics, University of California Irvine, Irvine, California, United States of America; 5 Vector-Borne Disease Control Center 2.3, Ministry of Public Health, Tak, Thailand; 6 Department of Social and Environmental Medicine, Faculty of Tropical Medicine, Mahidol University, Bangkok, Thailand; T D Medical College, INDIA

## Abstract

Despite Thailand’s progress under the 1-3-7 malaria elimination framework, border districts such as Tha Song Yang in Tak Province continue to experience persistent transmission due to high population mobility, geographic constraints, and health system challenges. Understanding how local health systems respond to these pressures is critical for sustaining malaria elimination in complex border settings. This mixed-methods study applied the World Health Organization’s Six Building Blocks framework to examine challenges and responses in malaria elimination in Tha Song Yang District. Qualitative data were collected through in-depth interviews with 24 key informants from district health offices, vector-borne disease units, malaria posts and clinics, hospitals, and local authorities. Quantitative data included household surveys assessing malaria-related knowledge, attitudes, and practices (n = 388), and secondary surveillance data on adherence to the 1-3-7 strategy from 2018 to 2022. Adherence to Day 1 and Day 3 activities improved steadily, reaching 96% and 100%, respectively, while Day 7 adherence declined sharply to 69% in 2022. The household survey showed high levels of malaria knowledge (75.77%) and positive attitudes toward prevention (94.59%), indicating that community awareness was strong despite ongoing transmission. Qualitative findings highlighted a surge of short-term migrants, workforce shortages, reduced domestic funding, and logistical barriers in remote areas as key constraints, while adaptive local responses—including local flexibility, committed leadership, and the use of the Malaria Information System and mobile communication platforms—helped sustain malaria control and surveillance activities. These findings demonstrate that malaria persistence in border areas is driven primarily by systemic and operational challenges rather than gaps in community awareness. Strengthening malaria elimination in similar border settings requires resilient health systems with sustained domestic financing, adaptive surveillance strategies, effective use of digital tools, and governance arrangements that account for population mobility and cross-border complexity.

## Introduction

Malaria remains a pressing global health issue, with estimated 263 million cases and 597,000 malaria deaths worldwide in 2023, particularly affecting low- and middle-income countries in Africa and Southeast Asia [1]. While overall progress has been made in reducing incidence, recent years have seen stagnation and even resurgence in some regions because of conflict, mobility, and weakened health systems [2,3]. In the Greater Mekong Subregion, including Thailand, cross-border movement and underserved populations pose ongoing challenges to elimination efforts [4,5].

Tha Song Yang District in Tak Province consistently reports the highest number of malaria cases in Thailand. Although the overall incidence steadily declined over the past decade, a resurgence was observed in 2022 and 2023, with reported cases increasing to 2,137 and 2,724, respectively [6]. The district shares a long and porous border with Kayin State, Myanmar, where malaria transmission remains substantially higher than in Thailand, particularly in remote conflict-affected areas with limited health service coverage [7,8]. This cross-border epidemiological disparity results in a continuous influx of imported cases into Tha Song Yang, which in turn seed local transmission in receptive communities. Both imported and secondary locally acquired cases contribute to persistent hotspots [9]. These dynamics are further intensified by the district’s mountainous geography, limited infrastructure, and high population mobility, including undocumented and transient migrant populations, which collectively undermine timely diagnosis, treatment, and follow-up [10–12].

The persistence of both imported and locally transmitted cases in Tha Song Yang underscores the need for rapid detection and containment to prevent onward spread. In response, Thailand has adopted the 1-3-7 surveillance and response framework as the cornerstone of its malaria elimination policy. Under this framework, all confirmed cases must be reported to the Malaria Information System (MIS) within 24 hours of diagnosis (Day 1); investigated within three days to verify the case and classify it as locally acquired or imported (Day 3); and addressed within seven days through foci investigation and tailored response measures based on the case investigation findings and area stratification for each index case (Day 7) [13].

In high-endemic and highly mobile border settings such as Tha Song Yang, effective execution of the 1-3-7 strategy is essential but frequently constrained by geographic isolation, limited infrastructure, and health system capacity. Inadequate mobile network coverage, poor road conditions, limited access to diagnostics and treatment in new settlements, and difficulties in following up with mobile populations continue to impede adherence to the protocol [13–15]. These vulnerabilities were further exacerbated during the COVID-19 pandemic, which disrupted routine malaria control activities and weakened community engagement [3]. Following the relaxation of pandemic-related restrictions, renewed cross-border movement—compounded by political instability in Myanmar—coincided with a resurgence of malaria in Tha Song Yang in 2022 [6].

A previous study conducted in this district documented that, despite operational challenges during the pandemic, the long-lasting insecticidal nets distribution system demonstrated resilience because of strong intersectoral collaboration, effective logistical management, and the support of an electronic database system [16].

Although numerous studies in Tha Song Yang District have examined malaria epidemiology and surveillance [17–19], behavior and social determinants [10,20,21], antimalarial drug efficacy [22,23], vector ecology [24–27], and vaccine development [28,29], there remains a critical gap in integrated health system analyses that assess how multiple system components interact to influence malaria elimination in a high-endemic border setting. In particular, limited evidence exists on how service delivery, workforce capacity, financing, information systems, and governance collectively shape the implementation of the 1-3-7 strategy under conditions of high mobility and geographic constraint. A comprehensive understanding of these dynamics is crucial for informing adaptive strategies, ensuring resource sustainability, and promoting health equity [30,31]. To address this gap, this study applies the World Health Organization’s Six Building Blocks framework [32,33] using a mixed-methods approach to examine systemic challenges and local responses to malaria elimination in Tha Song Yang District. By integrating qualitative insights with quantitative surveillance and community data, this study provides policy-relevant evidence to inform adaptive malaria elimination strategies in complex border settings.

Therefore, this study aimed to examine the systemic challenges and current responses to malaria elimination in Tha Song Yang District through a health systems perspective. Using a mixed-methods design, the research integrates qualitative data from key informant interviews with quantitative insights from household surveys on knowledge, attitudes, and practices (KAP) related to malaria prevention, as well as secondary surveillance data from MIS. The analysis was structured around the WHO’s Six Building Blocks of Health System Strengthening to capture operational strengths, identify critical bottlenecks, and explore the interactions across components.

Findings from this study can inform context-specific improvements to subnational malaria elimination strategies, particularly in high-endemic and resource-constrained settings. The lessons learned from Tha Song Yang may also be applicable to other vulnerable border areas facing similar geographic, demographic, and health system constraints.

## Materials and methods

### Study design and setting

This study employed mixed-methods approach, integrating qualitative and quantitative components. The qualitative component explored stakeholder perspectives on malaria elimination, including implementation and experiences operational challenges of the 1-3-7 strategy. The quantitative component included (1) a retrospective review of adherence to the 1-3-7 surveillance and response protocols from 2018 to 2022, and (2) a household survey assessing KAP regarding malaria prevention.

**Fig 1:**
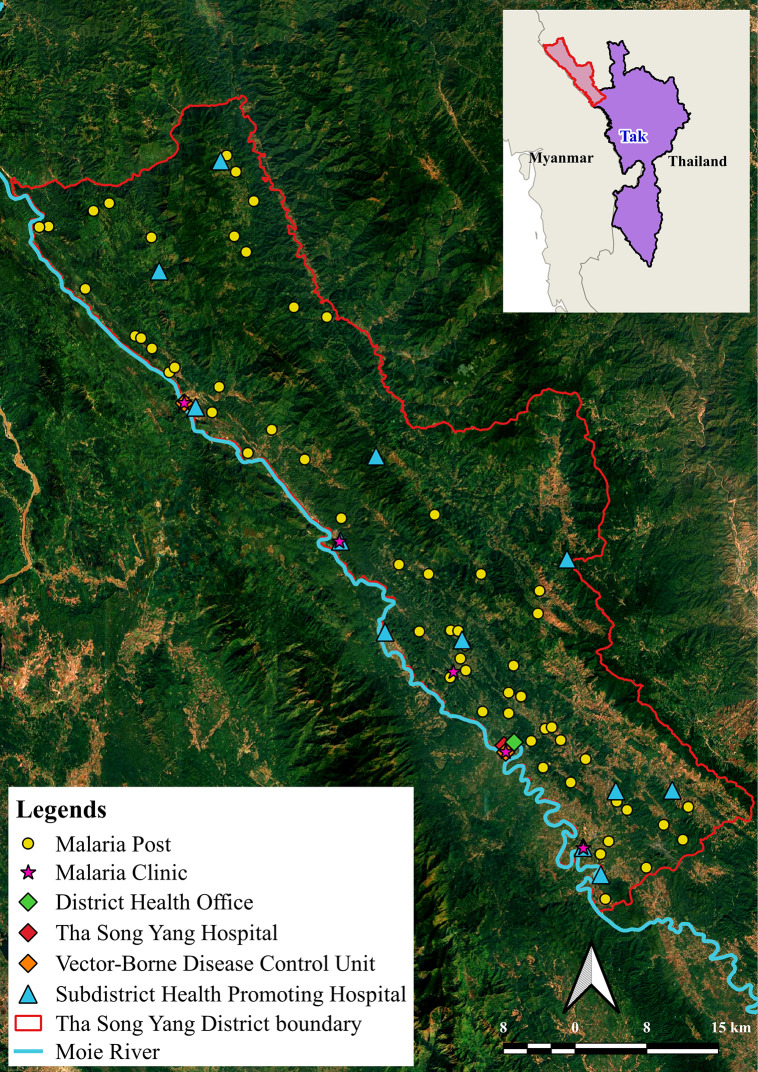
Study area and malaria-related locations in Tha Song Yang District, Tak Province, Thailand. The map was created by the authors using QGIS software. Administrative boundary shapefiles were obtained from the Humanitarian Data Exchange (https://data.humdata.org/dataset/cod-ab-tha) under a Creative Commons Attribution (CC BY) license. All point locations and thematic data were generated by the authors.

The study was conducted in Tha Song Yang District, Tak Province, Thailand, a border district with historically high malaria transmission and a large population of mobile and migrant individuals [10,12]. Tha Song Yang lies along the Moei River, directly bordering Myanmar in the northwestern region of the province. The district covers an area of 1,920.39 square kilometers and consists of six sub-districts. Malaria transmission follows a distinct seasonal pattern, with peaks typically occurring during the rainy season from May to October, coinciding with increased vector density and human mobility [10]. Health service delivery for malaria in the district is supported by a network of facilities and surveillance units strategically distributed across its sub-districts. As shown in Fig 1, this includes a district hospital, Subdistrict Health Promoting Hospitals (SHPHs), Malaria Posts (MPs), and Malaria Clinics (MCs) and Vector-Borne Disease Control Unit (VBDU) serving as the focal point for surveillance and response coordination. This spatial distribution of services reflects the need to provide both accessible community-based diagnosis and treatment, and centralized technical oversight to manage case detection, reporting, and foci response in a geographically challenging and highly mobile population setting.

In the Tha Song Yang context, the 1-3-7 surveillance and response strategy was implemented through a coordinated network of health actors. Day 1 (case detection and notification within one day) is primarily undertaken by MPs and MCs, which diagnose cases, initiate treatment, and report through the MIS or secure instant messaging platforms such as LINE application. SHPHs and Tha Song Yang Hospital also contribute to case confirmation and reporting, relaying data to the VBDU. Day 3 (case investigation and classification within 3 days) is conducted by VBDU surveillance teams, who interview patients to determine the source and classify cases as locally acquired or imported. Day 7 (foci investigation and response within 7 days) is also led by the VBDU, involving reactive case detection, environmental assessment, and targeted vector control in areas surrounding the index case’s residence.

### Participants and sampling

For the qualitative component, purposive sampling was used to select 24 key informants with direct operational role in malaria control in Tha Song Yang District. These included personnel from the VBDU (n = 4), District Health Office (DHO, n = 4), SHPHs (n = 4), MPs (n = 6), MC (n = 2), Local Administrative Organizations (LAOs, n = 2), and Tha Song Yang Hospital (n = 2). Participants consisted of 14 males and 10 females with age ranged between 36–55 years old. The VBDUs and DHOs are district-level entities responsible for coordinating and implementing vector-borne disease prevention and control activities. MPs and MCs are local malaria service units responsible for diagnosis and treatment. SHPHs represent primary care facilities within subdistricts.

For the quantitative component (KAP regarding malaria prevention), the sample size was calculated using the Statulator, online sample size calculator, for one-sample comparison of a proportion to a hypothesized value [34] A previous study conducted in malaria-endemic areas of Myanmar reported that 38.4% of respondents had good malaria knowledge [35]. For a 95% confidence level, and with an expected 10% non-response rate, the final sample size was set at 400. Villages were randomly selected from each subdistrict using the MIS database provided by the Department of Disease Control (DDC), Ministry of Public Health [6]. Within each selected village, the number of participants was determined using probability proportional to size. Subsequently, households in each selected village were chosen using systematic sampling. Eligible participants were household heads or adult household representatives, aged 18 years or older, who were willing to provide informed consent. Individuals were excluded if they had diagnosed cognitive impairment. The primary respondent was the head of household or representative, as identified through the household listing. If the selected individual was unavailable or declined to participate, the adjacent household to the right approached as a replacement.

### Data collection

Data was collected between January and April 2023. Prior to participation, all respondents were provided with an information sheet, and written informed consent was obtained. In-depth interviews were conducted with staff from seven key organizations involved in the implementation of malaria elimination efforts. To ensure that the essential components of the health system were comprehensively explored, the interview guide was structured around the WHO’s Six Building Blocks for Health System Strengthening including (1) service delivery, (2) health workforce, (3) health information systems, (4) medical products, vaccines, and technology, (5) health financing, and (6) leadership and governance [32]. Interviews were conducted in Thai, either face-to-face or via secure online platforms, and typically lasted between 45 and 60 minutes.

Additionally, secondary data on the implementation of the 1-3-7 surveillance and response activities from 2018 to 2022 were obtained from the Malaria Information System (MIS) of the Department of Disease Control. The aggregated surveillance data used for analysis were extracted from official MIS reports and are provided as a supplementary dataset (S1 Table). To assess community resident’s KAP related to malaria prevention, a structured questionnaire was administered to a sample of household representatives. The knowledge section consisted of 11 true-or-false items, with scoring categorized into three levels based on Bloom’s criteria [36]. The attitude section comprised 16 items using a 5-point Likert scale, categorized into three levels based on Best’s criteria [37]. The practice section included 7 questions assessing the use of various mosquito preventive measures. The questionnaire was developed through a literature review and showed good content validity (IOC = 0.75–0.96) and internal consistency (Cronbach’s α = 0.87; KR-20 = 0.90) based on pilot testing in a comparable village. The survey was conducted by a trained research team fluent in both Thai and Karen languages to ensure effective communication with ethnolinguistically diverse participants. Conceptual equivalence across languages was ensured through forward–backward translation by bilingual researchers. Standardized interviewer training and field supervision minimized interviewer variability, and private interviews with neutral wording were used to reduce response bias.

### Data analysis

All qualitative interviews were transcribed verbatim and analyzed using thematic analysis. The initial coding was guided by the WHO’s Six Building Blocks of Health System Framework, with additional themes emerging inductively from the data. Two independent researchers conducted coding and thematic validation to ensure analytical rigor and reduce potential bias. Discrepancies were resolved through discussion until consensus was reached.

For the quantitative component, data were entered and cross-validated in Microsoft Excel before being imported into STATA version 15 (licensed to the Faculty of Public Health, Thammasat University, Thailand) for analysis. A total of 400 households were targeted, 388 questionnaires were complete and included in the final analysis. Descriptive statistics were calculated, including means and standard deviations for continuous variables and frequencies with percentages for categorical variables. The quantitative results were used to complement and triangulate the qualitative findings, offering a comprehensive understanding of the implementation of malaria elimination program in the study setting.

### Ethics statement

The study protocol was conducted in accordance with the Declaration of Helsinki and was approved by the Institutional Review Board of Boromrajonani College of Nursing, Nakorn Lampang, Thailand (No. E2565-098 with a date of approval of 16 August 2022).

## Results

### Service delivery

**Fig 2:**
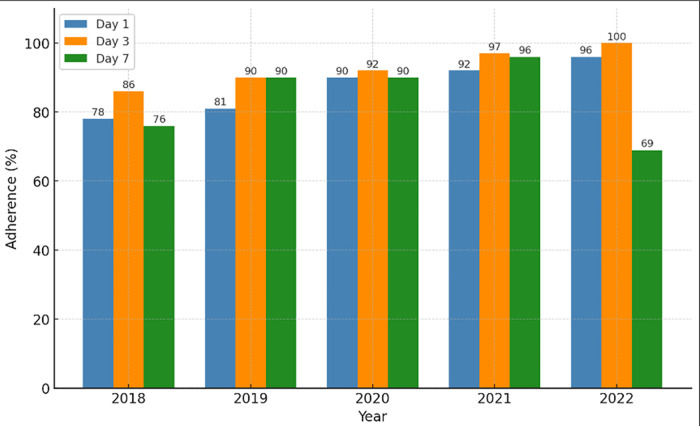
Adherence to the 1-3-7 surveillance and response protocols (2018–2022).

Analysis of surveillance data showed a consistent improvement in early case notification and investigation. Adherence to Day 1 increased from 78% to 96%, while Day 3 adherence improved from 86% to 100% between 2018 and 2022. In contrast, adherence to Day 7 protocols, which involve foci investigation and response, remained high from 2019 to 2021 but declined sharply from 96% in 2021 to 69% in 2022 (Fig 2).

Qualitative findings revealed multiple operational barriers affecting service delivery. Poor mobile network coverage in remote mountainous villages delayed Day 1 case notification, particularly from MP units. Travel constraints during the rainy season, including impassable roads, further hindered follow-up and foci investigation. Adherence to treatment and follow-up was especially challenging among short-term migrant patients (residing in Thailand for less than six months), many of whom were undocumented and highly mobile, making continued monitoring difficult. Even among Thai and long-term migrant patients, medication adherence follow-up at days 60 and 90 was difficult to complete.

Delays in foci investigation were frequently reported in relation to insufficient staffing and travel barriers, which prevented completion of Day 7 activities within the required timeframe. In 2022, these challenges coincided with a sharp increase in malaria cases (S2 Table), reduced budgets for malaria control, increased migration from Myanmar, and operational disruptions related to the COVID-19 pandemic.

*“Our MPs are located in an area without mobile network coverage. When there is a patient case, we have to travel to an area with network to report case notification. However, during the rainy season, traveling to that area becomes difficult.”* (35-year-old female, MP staff)

*“Tha Song Yang District has a high malaria burden, which overwhelms the available staff and prevents us from completing foci response within the 7-day target.”* (42-year-old male, VBDU staff)

**Fig 3:**
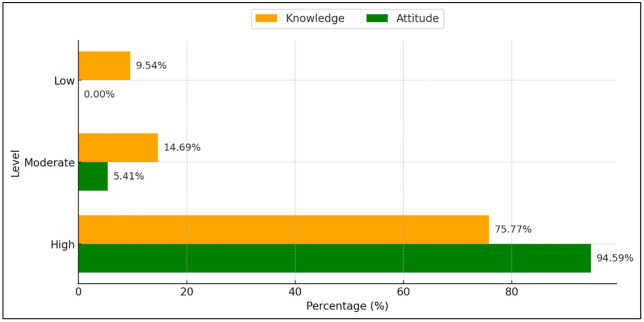
Knowledge and attitude regarding malaria prevention (n = 388).

**Fig 4:**
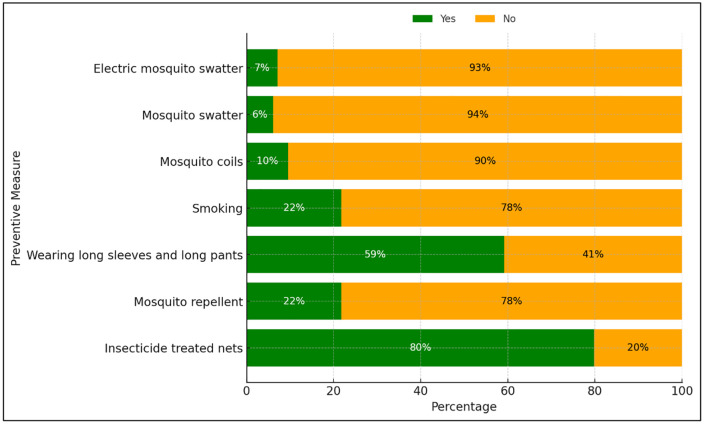
Mosquito prevention behaviors (n = 377).

Beyond surveillance, routine malaria prevention activities—including health education, IRS, distribution of long-lasting insecticidal nets, and provision of repellents—were implemented across the district. The household survey achieved a response rate of 97.0% and showed that 75.77% of participants had high malaria knowledge and 94.59% demonstrated positive attitudes toward malaria prevention (Fig 3). In addition, 97.16% of respondents (n = 377) reported practicing at least one mosquito prevention behavior, most commonly the use of insecticide-treated nets (Fig 4). The anonymized quantitative data from the household KAP survey are provided as supplementary files (S3 Table)

Recent policy shifts have narrowed IRS implementation from wide-scale spraying in active and residual non-active foci to case-based spraying around patient residences. Some informants perceived that reduced IRS coverage may have limited vector control intensity.

*“Restricting chemical spraying to areas around patient’s homes may have contributed to the resurgence of malaria cases, as vector control activity is not as intensive as before.”* (45-year-old male, VBDU staff)

Informants reported that malaria cases increased after COVID-19 movement restrictions were relaxed in 2022, prompting surveillance teams to extend working hours into the evenings and expand case detection in refugee camps.

*“Internal conflict in Myanmar increased malaria cases in Tha Song Yang District because many migrants settled along the Thai border and sought free treatment in Thailand.”* (48-year-old male, Tha Song Yang Hospital staff)

While malaria diagnostic and treatment services were generally accessible through MPs and MCs, newly formed migrant settlements along the border often lacked service coverage.

*“MP and MC units should be established in newly formed border villages to ensure all residents could access to malaria diagnosis and treatment services.”* (47-year-old male, DHO staff)

### Health workforce and collaboration

The VBDU surveillance team plays a central role in implementing the 1-3-7 strategy; however, workforce shortages, particularly during peak transmission periods, remain a major constraint. SHPH and MP staff frequently assist with blood testing and health education, while non-governmental organizations such as World Vision provide additional support.

*“We request SHPH and MP staff to help with blood testing, but if the target area is too far away, they cannot always participate.”* (45-year-old male, VBDU staff)

During the 2022 surge, additional personnel from Tha Song Yang Hospital and the Vector-Borne Disease Control Center (VBDC) were deployed to support surveillance and response. Coordination between provincial authorities and counterparts in Myanmar also facilitated cross-border information sharing.

*“Malaria cases surged after post COVID-19 overwhelmed our surveillance team. The VBDC sent two surveillance teams to assist in conduction the 1-3-7 strategy.”* (45-year-old male, VBDU staff)

Community and multi-sector collaboration further supported malaria elimination. Local leaders, border patrol police, military units, and schools contributed to data sharing, logistics, and health education.

*“VBDU staffs can request community leaders for data of malaria cases and their neighbor or seek military transport for remote areas. Border patrol schools integrate malaria education into their curriculum and assist student with medication adherence.”* (45-year-old male, VBDU staff)

*“We share information of malaria situation and educate the public at the meetings held every month with local leaders, teachers, and soldiers. In some year, we conduct malaria-dengue campaign in the high risk villages”* (50-year-old female, SHPH staff)

DDC is undergoing a policy shift, restructuring operation by transferring the responsibility of the 1-3-7 strategy from VBDU to SHPH staff and surveillance and rapid response team from DHO, which may result in a reduction of VBDU staff positions in the future.

*“With national policy shifting malaria work to SHPHs, VBDU staff positions may decline, but no concrete transition plan is in place yet in Tha Song Yang District.”* (45-year-old male, VBDU staff)

Because of the insufficiency of staff conducting malaria elimination activities (S2 Table), LAOs were encouraged to play a role in malaria elimination activities according to DDC policy. However, they have not played a significant role in that work yet. Most of LAO staff in Tha Song Yang District are willing to engage in the malaria elimination activities.

*“If LAO need to take on malaria work, they must be allocated more staff. However, our organization has sufficient staffs and is ready to participate if requested.”* (52-year-old male, LAO staff)

The officer responsible for malaria elimination activities in Tha Song Yang District receives annual refresher training on malaria elimination activities. They also exchange useful information, obstacles, and solutions while training. Additionally, a monthly meeting among Tha Song Yang Hospital, DHO, VBDU, and SHPHs take place to exchange knowledge and integrate disease control efforts.

*“Collaboration is key to our success in malaria work, but frequent staff rotations and retirements disrupt continuity.”* (48-year-old male, Tha Song Yang Hospital staff)

### Health financing

Malaria elimination activities in Tha Song Yang District relied predominantly on external funding, with approximately 90% from the Global Fund and 10% from domestic sources, mainly the DDC. Over time, declining DDC funding reduced support for mobile diagnostics, health education, travel allowances, supervision, and training for malaria volunteers. Moreover, monthly compensation for MP officers is only 90 USD, which is insufficient for livelihood, and the cancelation of travel allowances has increased their financial burden.

*“The budget allocated by DDC has decreased compared to the past due to the reduced prioritization of the disease, both numbers of patients and deaths have declined.”* (45-year-old male, VBDU staff)

*“We have to attend the meeting once every two months at the DHO. However, our MP units are located far from the DHO, so we have to incur significant travel costs and spend a lot of time on the journey.”* (35-year-old female, MP staff)

Local Administrative Organizations (LAOs) provided limited financial support, primarily for bed net impregnation and IRS since 2020. During the 2022 surge, supplemental funding was requested from DDC and the Global Fund to support outbreak control.

*“Normally, we are not able to request budget adjustments DDC, but due to the surge in malaria cases in 2022, the department provided us with additional funds for outbreak control as a special exception.”* (45-year-old male, VBDU staff)

### Access to essential medicines and technologies

Periodic shortages of antimalarial medicines and diagnostic kits occurred, particularly during high transmission periods. Although local sharing of supplies among VBDU, hospitals, and DHO helped mitigate shortages, gaps persisted.

*“We were allocated less Primaquine than requested because it was unavailable in the material warehouse.”* (48-year-old male, Tha Song Yang hospital staff)

*“We managed medical supplies locally, among VBDU, hospitals, and DHO. We share or borrow medicines or testing kits if these are insufficient. During high transmission periods, especially in 2022, shortages of these supplies obstructed outbreak control.”* (45-year-old male, VBDU staff)

Insufficient supplies delayed diagnosis and treatment, especially when MP units ran out of test kits, forcing patients to travel farther for care.

### Health information system

The consolidation of malaria data from various sources to the same database system, a national web-based MIS, can enhance the effectiveness of malaria elimination intervention in Tha Song Yang District. The system operates even in areas without mobile network coverage, ensuring continuous recording data in remote locations. The availability of real-time data has enabled the staff to make a decision, plan, and execute interventions more effectively.

*“VBDU, DHO, and hospital staff can use data from MIS for planning and decision-making in malaria elimination activities—such as target analysis, health education, public communication, and implementing the 1-3-7 strategy.”* (47-year-old male, DHO staff)

### Leadership and governance

Leadership and governance in malaria elimination efforts in Tha Song Yang District face significant challenges because of declining staff numbers, decreasing number of MP units, reduced budget for malaria elimination activities, and intermittent equipment supplies, posing challenges to malaria elimination efforts.

*“Malaria is a disease that, once contracted, is not normally fatal and can usually be completely cured. When cases decline, so does the interest, lowering the disease’s priority.”* (48-year-old male, Tha Song Yang Hospital staff)

Staff emphasized that successful malaria elimination requires increased budget allocations, sufficient human resources, greater involvement from communities, private sector, non-governmental organizations, and public agencies, consistent provision of essential equipment, and strengthened efforts focused along the border areas. Nonetheless, disease control remains challenging, as many temporary Myanmar residents are reluctant to seek treatment due to fears of deportation, which was reported by staff as a challenge for timely diagnosis.

## Discussion

This study assessed the challenges and responses of malaria elimination strategies in Tha Song Yang District through the lens of the World Health Organization’s Six Building Blocks of the Health System framework. The findings highlight both the district’s strengths and the persistent systemic and contextual challenges that continue to impede sustained progress toward malaria elimination.

### Progress and limitations of the 1-3-7 strategy implementation

The 1-3-7 strategy remains central to Thailand’s malaria elimination efforts, supported by trained personnel, cross-sector collaboration, and the effective use of MIS [38]. In Tha Song Yang, adherence to Day 1 and Day 3 protocols steadily improved from 2018–2022, reflecting progress in early detection and investigation. However, Day 7 adherence dropped to 69% in 2022, which may be partly explained by increased post-COVID-19 mobility and the influx of undocumented and short-term migrants from Myanmar [1,4,39]. Respondents reported that Day 7 failures primarily occurred when index cases were unavailable for follow-up due to cross-border movement or relocation shortly after diagnosis, preventing timely foci delineation, and were further compounded by limited surveillance capacity during the surge in cases. These groups, often residing in remote and informal settlements, face legal and logistical barriers that delay care and hinder follow-up, contributing to continued transmission risks [40,41].

Compounding this issue are recent shifts in vector control policy from wide-scale IRS in active foci and non-active foci areas to targeted, case-based spraying. While more efficient, this approach may miss high-risk zones with mobile and undocumented populations. Experiences from other Greater Mekong Subregion borders suggest that narrowing IRS coverage without enhanced surveillance risks leaving hidden transmission foci [1,42]. It is important to note that this observation does not imply a direct causal relationship between reduced IRS coverage and malaria resurgence; rather, it reflects key informant perspectives and epidemiological plausibility in a highly receptive transmission setting characterized by population mobility.

More broadly, although stronger cross-border collaboration is widely recognized in literature as essential for sustainable malaria elimination, its implementation in some border contexts remains challenging. In areas affected by ongoing conflict, fragmented governance structures and rapidly changing administrative conditions have been reported as barriers to coordinated cross-border action. These contextual constraints, documented in previous regional studies, underscore the need for adaptive, locally resilient strategies that do not rely solely on formal cross-border governance mechanisms.

### Service delivery in challenging border geography and vulnerable populations

Tha Song Yang’s rugged terrain, poor infrastructure, and weak mobile network coverage significantly hinder timely malaria service delivery. Delays in case reporting by MPs, who must travel to areas with signal, compromise the responsiveness of the 1-3-7 strategy and coordination with district-level teams [43]. During the rainy season, access to remote villages is further obstructed by flash floods and landslides, delaying case investigations and focal responses [44,45]. These logistical challenges mirror those in other Greater Mekong Subregion border areas, where service continuity depends on adaptive delivery mechanisms [4]. Bridging these gaps requires investment in communication networks, year-round transport infrastructure, and mobile health teams equipped for offline diagnostics Equally important is tailoring services to diverse populations, particularly undocumented migrants who often face legal barriers to accessing formal health services, as observed in this study. In addition, existing literature highlights that cultural and linguistic barriers may further constrain service utilization among migrant populations in border settings [41]. Taken together, these challenges underscore the need for a universal, flexible, and culturally sensitive service delivery model that can adapt to heterogeneous and mobile populations.

A universal, flexible, and culturally sensitive service delivery model remains critical to ensure equitable malaria elimination in these hard-to-reach settings. However, new migrant settlements in remote border areas may remain beyond the reach of existing MP and MC coverage. This creates an emerging equity gap in access to timely diagnosis and treatment, risking the development of hidden transmission zones. Proactive efforts to reach these displaced populations, through improved diagnostics and strengthened surveillance, are essential to sustaining elimination gains [46].

### Health workforce and intersectoral collaboration

A skilled and stable workforce is essential for effective malaria surveillance. In Tha Song Yang, staff shortages—particularly in VBDU, SHPHs, and MPs—significantly constrained outbreak response, especially in remote areas. During peak seasons, limited personnel were stretched thin, leading to operational delays. Factors such as geographic isolation, safety risks near conflict zones, and limited financial incentives further hindered staff recruitment and retention [47,48]. While temporary collaboration with non-governmental organizations and civil society helped bridge service gaps in hard-to-reach communities, such support was often project-based, lacked long-term continuity, and remained outside formal health system structures. This reliance risks fragmentation and undermines accountability in surveillance and response.

The Ministry of Public Health’s plan to shift malaria responsibilities to primary care units has the potential to improve service integration. However, evidence from national policy documents and previous studies suggests that such transitions may disrupt service continuity if not accompanied by adequate workforce planning, training, and institutional preparedness [49,50]. In high-endemic, logistically challenging districts like Tha Song Yang, decentralization must be matched with investments in human resource development and sustained multisectoral coordination.

### Financial sustainability

While Tha Song Yang continues to receive malaria support from external donors such as the Global Fund, declining domestic funding, particularly from the DDC, has disrupted surveillance, training, and coordination activities. These constraints might further be exacerbated by the national policy shift toward integrating malaria activities into SHPHs in the absence of a clear transition plan, creating uncertainty around workforce roles, budgeting responsibilities, and operational continuity, particularly as VBDU staff positions are expected to decline. Field operations, including case investigation, reactive detection, and outreach, were constrained by limited travel support, delayed training, and reduced logistical resources, with some community workers relying on personal funds to sustain essential activities.

Together, these financial and governance gaps weakened core components of the 1-3-7 strategy and may have contributed to the 2022 resurgence. WHO has cautioned that funding gaps threaten elimination gains, especially during transitional phases [51], and previous studies emphasize the importance of strengthening domestic and subnational financing in donor-dependent settings [42]. In Thailand, however, LAOs remain underutilized, with contributions largely confined to short-term project support. Strengthening financial sustainability will require clearer fiscal decentralization, protected local budget lines for malaria activities, and stronger accountability mechanisms to embed malaria financing within routine local governance, particularly in high-burden border areas.

### Medicine and diagnostic supply chain

Although local coordination between VBDU, hospitals, and SHPHs enables sharing of malaria diagnostics and drugs, national-level procurement remains a critical bottleneck. Shortages of key commodities, especially primaquine, caused by limited domestic production and regulatory hurdles compromise the treatment of *P. vivax* and relapse prevention [42,52]. During peak transmission periods, stockouts of rapid diagnostic test and antimalarials delay timely diagnosis and case management, weakening 1-3-7 effectiveness. Stakeholder interviews and national reviews point to inadequate forecasting and centralized procurement processes that lack agility in responding to urgent local needs, especially in remote border districts [53]. Furthermore, the low commercial value of essential malaria drugs reduces production incentives, contributing to recurrent supply gaps. These disruptions can lead to incomplete treatment, delayed outbreak control, and declining community trust in public health services—particularly among mobile populations [54]. Addressing these challenges will require decentralizing procurement systems, strengthening coordination between national and local units, and investing in local buffer stockpiles.

### Health information system

Thailand’s MIS plays a key role in supporting the 1-3-7 strategy through real-time case tracking, foci investigation, and microstratification—even in offline settings. Its evolution from the earlier eMIS platform has improved case management and decision-making, particularly in remote and border areas, aligning with WHO recommendations for digital, granular surveillance systems in elimination-phase settings [13,54,55]. Despite these strengths, field-level use of MIS remains limited. Barriers include poor internet connectivity, low data literacy, and continued reliance on paper-based tools in some areas. These constraints reduce the system’s effectiveness for timely response and local planning. To optimize MIS utility, efforts should focus on building analytic capacity, decentralizing data use, and promoting MIS-driven microplanning at the subdistrict level.

### Local governance and continuity

Sustained malaria elimination requires stable governance, political will, and strong local leadership. In Tha Song Yang, structural transitions such as staff retirement, rotation, and the transfer of responsibilities from vertical programs (e.g., VBDU) to primary care units have raised concerns about program continuity. Without parallel investments in workforce development and institutional readiness, these changes may disrupt operations [56]. Moreover, LAOs have contributed only minimally, with most support being short-term and project-based, lacking alignment with national malaria goals [57,58]. This mirrors broader challenges in decentralized systems where local bodies often lack budgets, technical expertise, and clear mandates to support disease control [59,60]. Strengthening subnational governance through clearer roles, financial devolution, and community involvement is critical to building resilient, locally owned malaria responses in high-risk districts like Tha Song Yang.

### Limitations

First, adherence indicators derived from MIS data may be affected by reporting delays and incompleteness in remote border areas and should therefore be interpreted as programmatic estimates rather than precise operational measures. Second, the qualitative findings, based on purposive sampling in a high-endemic border district, are context-specific and not statistically generalizable, but may be analytically transferable to similar malaria-endemic border settings. Third, the KAP survey was cross-sectional, and qualitative interviews were conducted shortly after the 2022 malaria resurgence; therefore, the findings reflect a temporal snapshot of health system performance during a period of heightened pressure and may emphasize outbreak response and post-pandemic mobility challenges rather than longer-term trends.

## Conclusion

This study provides a health systems perspective on malaria elimination in Tha Song Yang District, a high-endemic border area characterized by geographic isolation, population mobility, and resource constraints. While the 1-3-7 strategy has strengthened early case detection and coordination, the findings illustrate how persistent challenges across service delivery, workforce capacity, financing, supply chains, and governance continue to shape implementation at the local level.

At the same time, the study highlights adaptive capacities within the local health system. Cross-sectoral collaboration among VBDUs, SHPHs, hospitals, and community networks, along with the use of digital tools such as the MIS and mobile communication platforms, supported continued surveillance and response despite operational constraints. These locally driven adaptations helped sustain core 1-3-7 activities, particularly timely reporting and investigation, during periods of heightened pressure.

Taken together, the findings underscore that malaria elimination in complex border settings is strongly influenced by local health system resilience and contextual factors. Rather than offering prescriptive policy solutions, this study contributes empirical evidence on how health system components interact in a high-burden border district. The insights generated may inform context-sensitive planning and implementation strategies in other settings facing similar challenges, while highlighting the importance of tailoring malaria elimination efforts to local realities.

## Supporting information

S1 Table Percentage of adherence to the 1-3-7 surveillance and response protocols (2018–2022). (XLSX)

S2 Table Number of malaria cases, active foci villages, and staffs in Tha Song Yang District. (DOCX)

S3 Table Household KAP survey. (XLSX)
