# Correction: Challenges and responses of malaria elimination in a high-endemic area along the Thai-Myanmar border: A health systems perspective

**DOI:** 10.1371/journal.pgph.0006446

**Published:** 2026-05-07

**Authors:** Sayambhu Saita, Daniel M. Parker, Kritsana Suk-uam, Suparat Phuanukoonnon, Kasama Pooseesod

The reference #18 is incorrect. The correct reference is:

Aung PL, Rachaphaew N, Sripoorote P, Htwe KZ, Suk-Aum K, Kaewkungwal J, Lawpoolsri S, Cui L, Sattabongkot J. Slide positivity, trends, and risk factors of febrile Plasmodium vivax malaria along the Thailand–Myanmar border, 2018–2023. Infectious Diseases of Poverty. 2025;14(1):82. https://doi.org/10.1186/s40249-025-01350-4 PMID: 40770656.
