# Supplementary material for: Challenges and responses of malaria elimination in a high-endemic area along the Thai-Myanmar border: A health systems perspective
Source: PLOS Glob Public Health. 2026 Apr 9;6(4):e0006286. doi: 10.1371/journal.pgph.0006286 (PMC13065034; doi:10.1371/journal.pgph.0006286)
Supplement: S2 Table — (DOCX) [file pgph.0006286.s002.docx]

S1 Table: Number of malaria cases, active foci villages, and staff in Tha Song Yang District.

|  | **Year** | | | | |
| --- | --- | --- | --- | --- | --- |
|  | **2018** | **2019** | **2020** | **2021** | **2022** |
| **Malaria cases** |  |  |  |  |  |
| National | 6,728 | 5,431 | 3,946 | 3,268 | 10,154 |
| Tha Song Yang (%) | 601 (8.93) | 420 (7.73) | 467 (11.83) | 471 (14.41) | 2,517 (24.79) |
| **Active foci villages** |  |  |  |  |  |
| A1 | 48 | 42 | 42 | 33 | 46 |
| A2 | 22 | 11 | 13 | 6 | 16 |
| **Staff** |  |  |  |  |  |
| No. MP staff | 74 | 74 | 74 | 74 | 74 |
| No. MC staff | 5 | 5 | 5 | 5 | 5 |
| No. VBDU staff | 13 | 13 | 13 | 13 | 13 |
